# Supplementary material for: Stochastic satisficing account of confidence in uncertain value-based decisions
Source: PLoS One. 2018 Apr 5;13(4):e0195399. doi: 10.1371/journal.pone.0195399 (PMC5886535; doi:10.1371/journal.pone.0195399)
Supplement: S1 Fig — We identified parameters that maximized the amount of reward accumulated by each of our models with the reward distribution from experiment 1, and examined the amount of reward collected by the models using these parameters over 100 repetitions. We also examined the rewards accrued by a model with full knowledge of the reward distribution (‘Omniscient’), and the actual amount of reward accrued by our participants. We found that all three models performed similarly, and accrued similar amount of reward. When the drifting mechanism was added (drift of the unchosen option towards the acceptability threshold) performance of all models decreased. All models did not accrue as much reward as the ‘Omniscient’ model, as all of them had to learn and adapt to a dynamic and changing environment. In addition, all models performed much better than our participants, indicating that participants’ behaviour was noisy, falling short of the optimal strategy. (PDF) [file pone.0195399.s001.pdf]

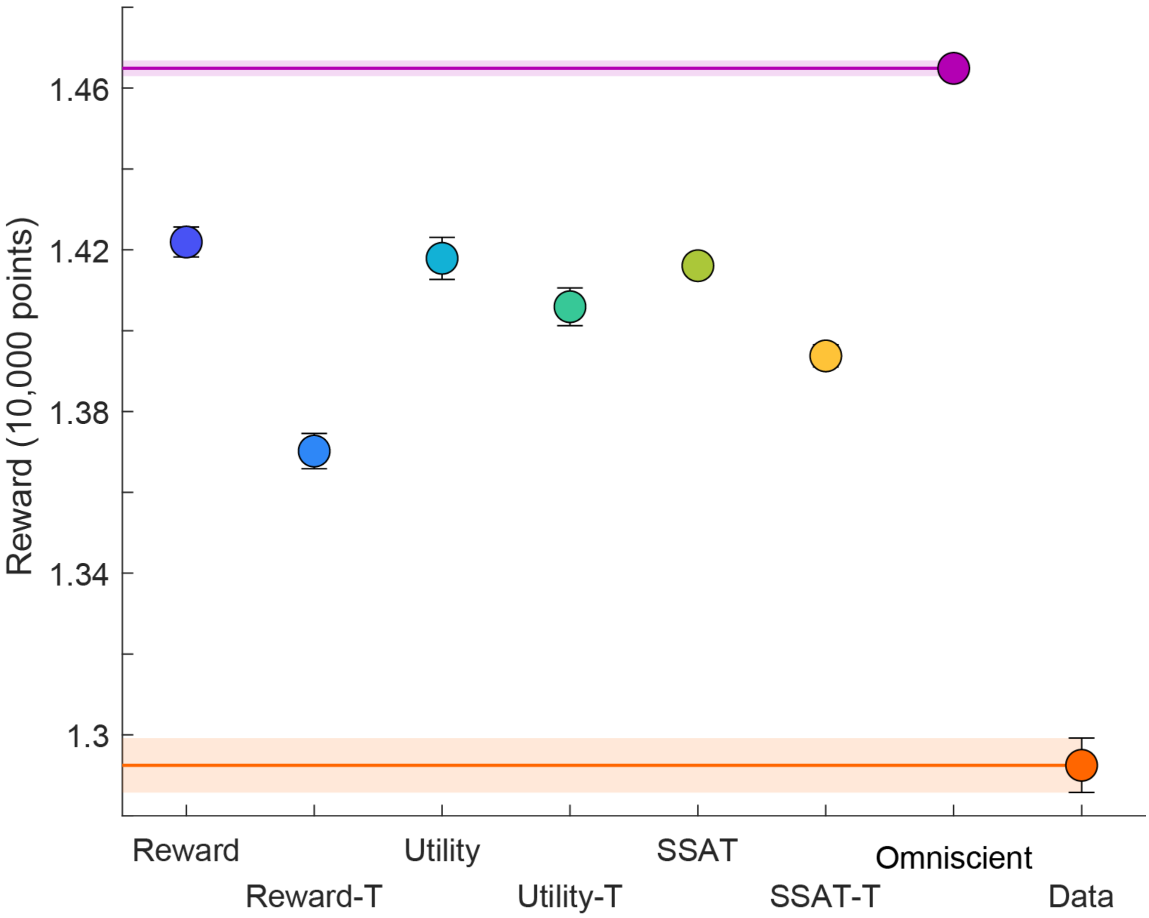

S1 Fig. Trial-by-Trial Optimality Analysis Experiment 1

We identified parameters that maximized the amount of reward accumulated by each of our models with the reward distribution from experiment 1, and examined the amount of reward collected by the models using these parameters over 100 repetitions. We also examined the rewards accrued by a model with full knowledge of the reward distribution ('Omniscient'), and the actual amount of reward accrued by our participants. We found that all three models performed similarly, and accrued similar amount of reward. When the drifting mechanism was added (drift of the unchosen option towards the acceptability threshold) performance of all models decreased. All models did not accrue as much reward as the 'Omniscient' model, as all of them had to learn and adapt to a dynamic and changing environment. In addition, all models performed much better than our participants, indicating that participants' behaviour was noisy, falling short of the optimal strategy.
