# Supplementary material for: Stochastic satisficing account of confidence in uncertain value-based decisions
Source: PLoS One. 2018 Apr 5;13(4):e0195399. doi: 10.1371/journal.pone.0195399 (PMC5886535; doi:10.1371/journal.pone.0195399)
Supplement: S2 Fig — We examined the differences in values assigned to the two options by each model in the four conditions of our experimental design. We used the reward distributions mean and variances in each condition, and varied the acceptability threshold (blue to yellow lines). We found that all models assigned higher values to the high mean reward option than to the low mean reward option in almost all the cases and conditions. A greedy decision maker would therefore be able to accumulate similar amount of rewards using each model. However, different models assigned different value differences in each condition. This means that a noisy decision maker (modeled using softmax) may be more likely to choose the low mean reward option in some conditions, according to the models’ predictions. (PDF) [file pone.0195399.s002.pdf]

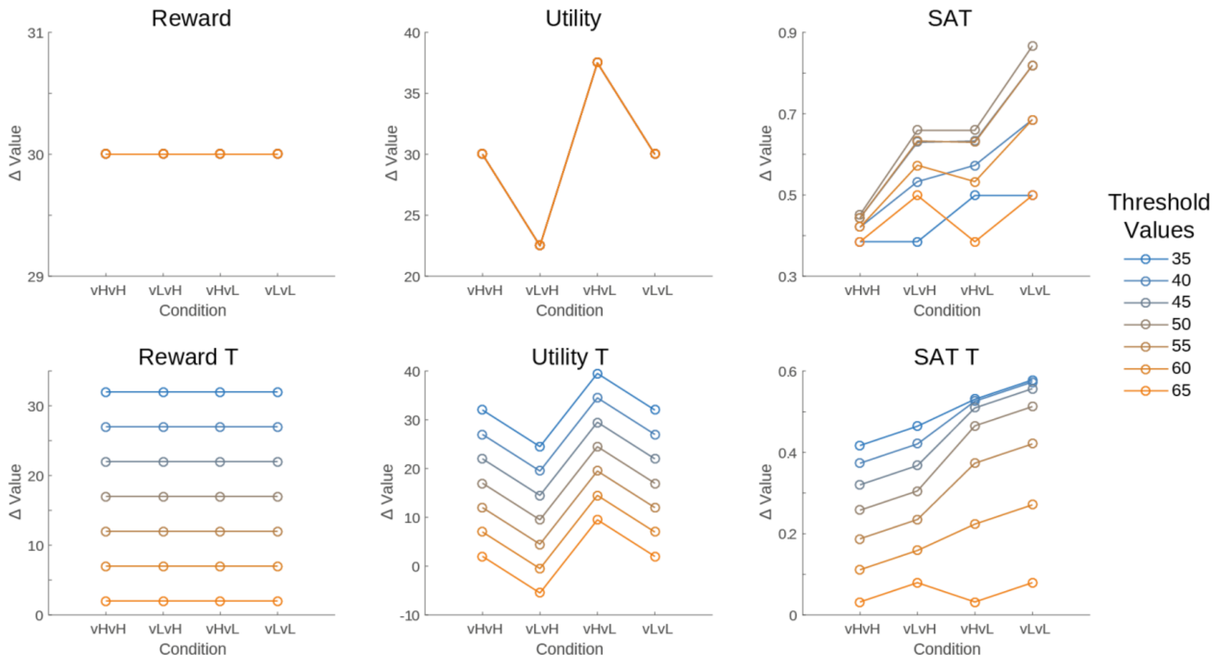

S2 Fig. Value-Differences Optimality Analysis in Experiment 1

We examined the differences in values assigned to the two options by each model in the four conditions of our experimental design. We used the reward distributions mean and variances in each condition, and varied the acceptability threshold (blue to yellow lines). We found that all models assigned higher values to the high mean reward option than to the low mean reward option in almost all the cases and conditions. A greedy decision maker would therefore be able to accumulate similar amount of rewards using each model. However, different models assigned different value differences in each condition. This means that a noisy decision maker (modeled using softmax) may be more likely to choose the low mean reward option in some conditions, according to the models' predictions.
