# Supplementary material for: Stochastic satisficing account of confidence in uncertain value-based decisions
Source: PLoS One. 2018 Apr 5;13(4):e0195399. doi: 10.1371/journal.pone.0195399 (PMC5886535; doi:10.1371/journal.pone.0195399)
Supplement: S3 Fig — (A) Trial-by-Trial frequency of choosing the good option across participants (grey line) and models estimations of probability of choosing the good option (coloured lines), averaged across participants (shaded areas represent SEM). (B) Models’ estimations were averaged between trials 10–25 in each block. The average choices made by participants is displayed in grey. The models lacking the drift-to-threshold mechanism (top row) showed less correspondence to the behavioural results. In addition, both ‘Utility’ models failed to replicate the low probability of choosing the good option in the vHvH condition (compared to vLvH condition), as they penalised both high and low mean options for variance in the same manner, whereas the SAT models penalised the good (high mean) option for variance, but promoted the bad option when its variance increased. The overall best fitting model, across all trials, was the ‘Reward T’. Error bars represent SEM. (PDF) [file pone.0195399.s003.pdf]

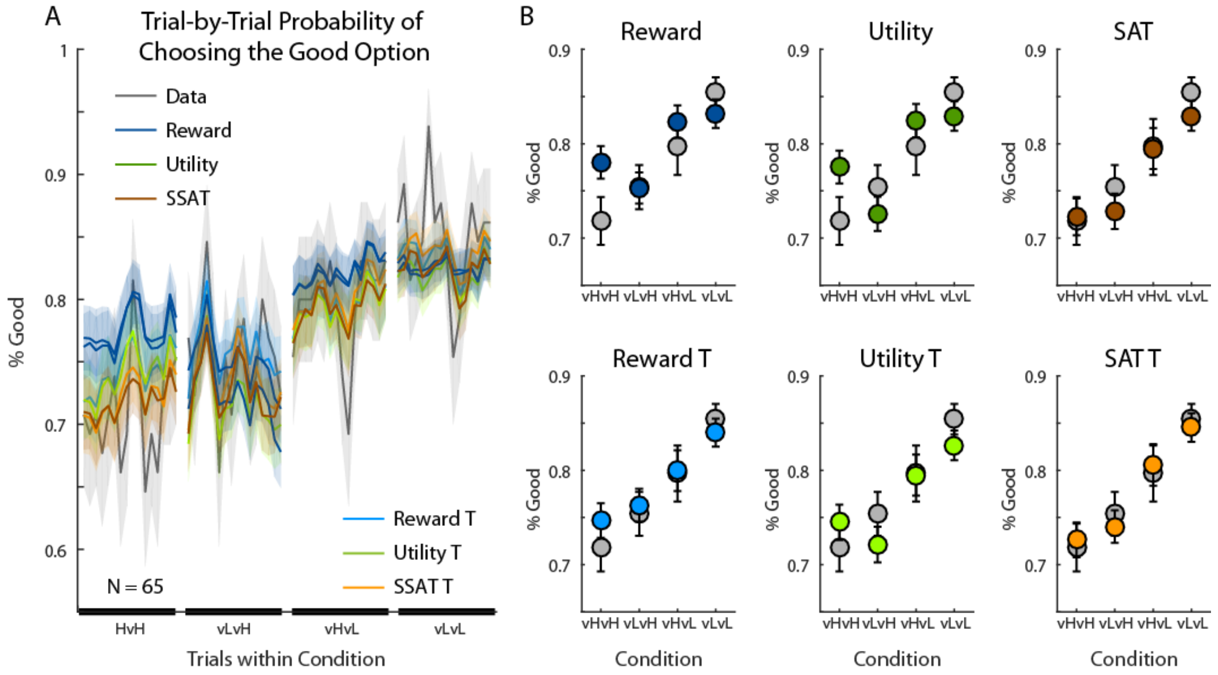

S3 Fig. Models fit to choices in experiment 1

(A) Trial-by-Trial frequency of choosing the good option across participants (grey line) and models estimations of probability of choosing the good option (coloured lines), averaged across participants (shaded areas represent SEM). (B) Models' estimations were averaged between trials 10-25 in each block. The average choices made by participants is displayed in grey. The models lacking the drift-to-threshold mechanism (top row) showed less correspondence to the behavioural results. In addition, both 'Utility' models failed to replicate the low probability of choosing the good option in the vHvH condition (compared to vLvH condition), as they penalised both high and low mean options for variance in the same manner, whereas the SAT models penalised the good (high mean) option for variance, but promoted the bad option when its variance increased. The overall best fitting model, across all trials, was the 'Reward T'. Error bars represent SEM.
