# Supplementary material for: Stochastic satisficing account of confidence in uncertain value-based decisions
Source: PLoS One. 2018 Apr 5;13(4):e0195399. doi: 10.1371/journal.pone.0195399 (PMC5886535; doi:10.1371/journal.pone.0195399)
Supplement: S4 Fig — We examined how many of the participants’ choices were best explained by each of our six models in both experiments (left panels), and how many participants’ confidence reports were best predicted by the models (right panels). We found that in Experiment 1 most of the participants’ choices were best explained by models that did not track reward variance, in line with the model comparisons we performed. In Experiment 2 choice responses were split between models that tracked variance and models that did not track variance. Best confidence ratings predictions were also distributed across participants. We found that in Experiment 1 most participants’ confidence reports were affected by variance, with half of the participants’ confidence reports best predicted by the SSAT or SSAT-T models. In Experiment 2 the picture was even more robust, with even greater share of the participants’ reports being affected by outcome variance. The distributions of confidence and choices were found to be different (Two-sample Kolmogorov-Smirnov test, Experiment 1: p = 0.0049, Experiment 2: p = 0.03). (PDF) [file pone.0195399.s004.pdf]

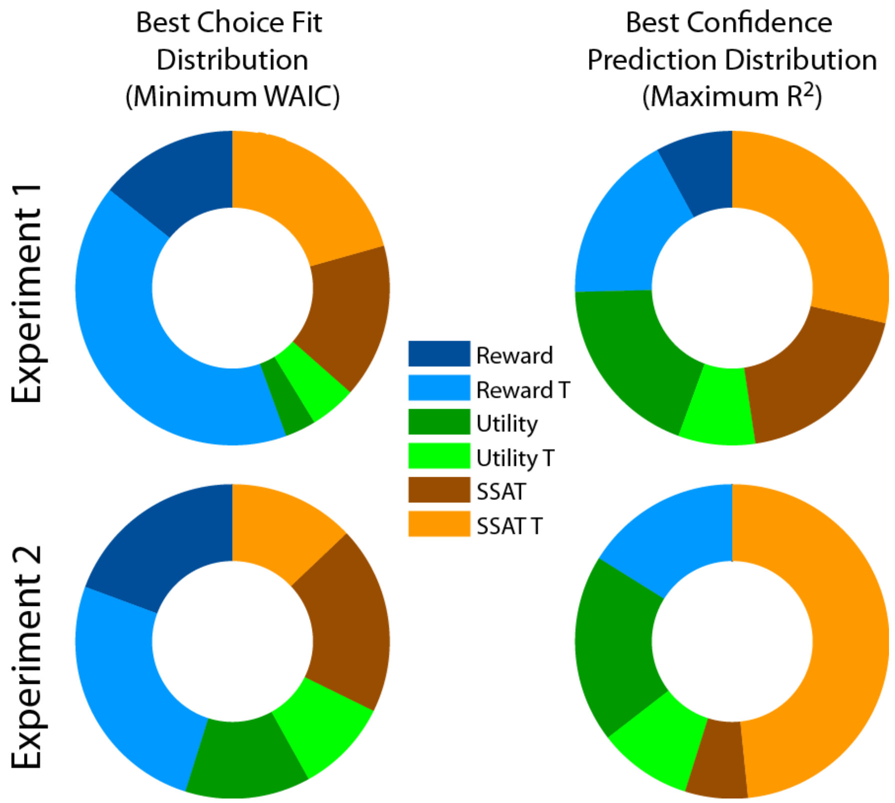

S4 Fig. Distribution of Best Model Fits Across Participants

We examined how many of the participants' choices were best explained by each of our six models in both experiments (left panels), and how many participants' confidence reports were best predicted by the models (right panels). We found that in Experiment 1 most of the participants' choices were best explained by models that did not track reward variance, in line with the model comparisons we performed. In Experiment 2 choice responses were split between models that tracked variance and models that did not track variance. Best confidence ratings predictions were also distributed across participants. We found that in Experiment 1 most participants' confidence reports were affected by variance, with half of the participants' confidence reports best predicted by the SSAT or SSAT-T models. In Experiment 2 the picture was even more robust, with even greater share of the participants' reports being affected by outcome variance. The distributions of confidence and choices were found to be different (Two-sample Kolmogorov-Smirnov test, Experiment 1: $p = 0.0049$, Experiment 2: $p = 0.03$).
