# Supplementary material for: Stochastic satisficing account of confidence in uncertain value-based decisions
Source: PLoS One. 2018 Apr 5;13(4):e0195399. doi: 10.1371/journal.pone.0195399 (PMC5886535; doi:10.1371/journal.pone.0195399)
Supplement: S6 Fig — (A) Trial-by-Trial confidence reports (grey line) and model predictions during each experimental condition are displayed, averaged across participants (shaded areas represent SEM). (B) Models’ predictions for confidence reports when choosing the good option (Top Row) and when choosing the bad option (bottom row). Predictions were averaged between trials 10–25 in each block. The average reports made by participants is displayed in grey. All models predicted higher confidence when choosing the good option than when choosing the bad option. Error bars represent SEM. (PDF) [file pone.0195399.s006.pdf]

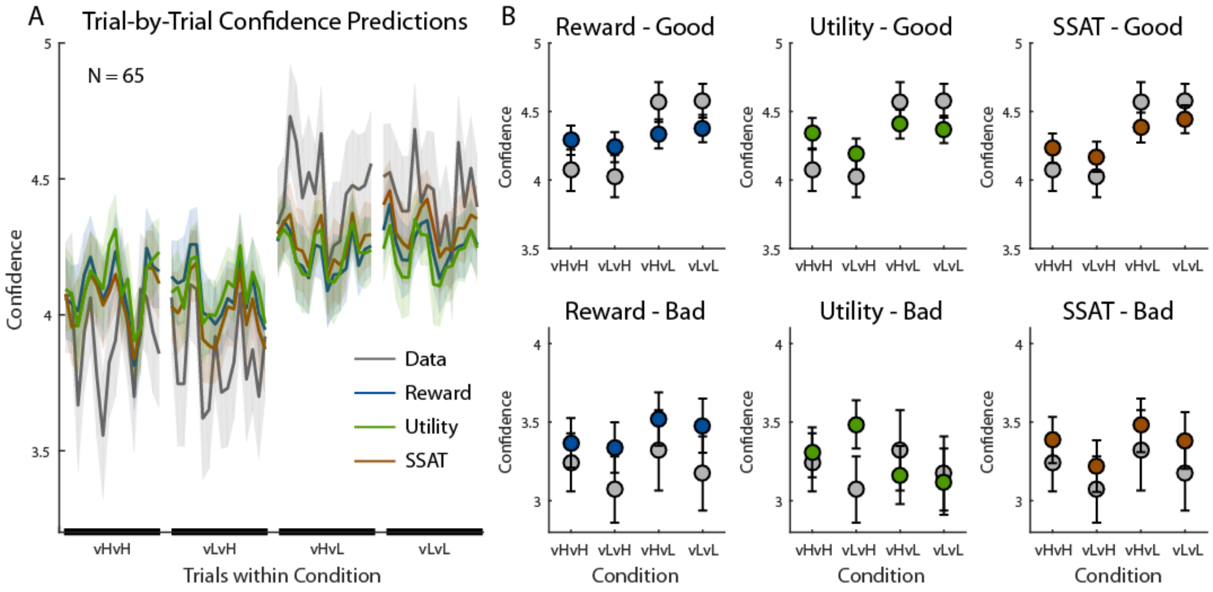

S6 Fig. Models predictions for confidence reports in experiment 1

(A) Trial-by-Trial confidence reports (grey line) and model predictions during each experimental condition are displayed, averaged across participants (shaded areas represent SEM). (B) Models' predictions for confidence reports when choosing the good option (Top Row) and when choosing the bad option (bottom row). Predictions were averaged between trials 10-25 in each block. The average reports made by participants is displayed in grey. All models predicted higher confidence when choosing the good option than when choosing the bad option. Error bars represent SEM.
