# Supplementary material for: Stochastic satisficing account of confidence in uncertain value-based decisions
Source: PLoS One. 2018 Apr 5;13(4):e0195399. doi: 10.1371/journal.pone.0195399 (PMC5886535; doi:10.1371/journal.pone.0195399)
Supplement: S7 Fig — To examine other utility functions we fitted a utility model which transforms the rewards in each trial according to the power utility function [1,2]: U(R)=R(1−γ)1−γ0≤γ<1 Where γ is the risk aversion factor–the closer it is to 1 the participant is more risk averse (and the closer the function is to log(r)). We used a model that learns from these transformed values, i.e. from utilities and not directly from the rewards, but was otherwise exactly the same as the ‘Reward’ model: {Qa(t+1)=Qa(t)+α(U(R(t))−Qa(t))Qb(t+1)=Qb(t) When option a is chosen, its value is updated according to the difference between its current value and the utility of the option’s current reward, with a learning rate α. Decision in each trial was then carried using a softmax rule. We fitted this ‘Power’ model to the choice data, and an additional ‘Power-T’ model which added the drift to threshold of the unchosen option mechanism: {Qa(t+1)=Qa(t)+α(U(R(t))−Qa(t))Qb(t+1)=Qb(t)+α(T−Qb(t)) We examined the fit of these models to the data and how well they predicted the confidence reports. We found that the ‘Power’ and ‘Power-T’ models performed very similarly to the ‘Reward’ and ‘Reward-T’ models respectively. Their WAIC values were: ‘Power’ 228.06 ± 67.46, ‘Power-T’: 214.51 ± 68.66, whereas the ‘Reward’ models WAIC values were: ‘Reward’ 226.55 ± 67.67, ‘Reward-T’ 214.57 ± 68.79. ‘Power-T’ was as good as our best model in explaining choice behaviour. We than examined how well the ‘Power’ models explained confidence reports. Again, they fared similarly to the ‘Reward’ models with linear fit (R2) of: Power’ 0.21 ± 0.22, ‘Power-T’: 0.21 ± 0.21, whereas the ‘Reward’ models WAIC values were: ‘Reward’ 0.21 ± 0.22, ‘Reward-T’ 0.21 ± 0.21. Finally, we examined the predicted confidence reports in the four condition blocks, and found that the patterns predicted by the ‘Power-T’ model were identical to the pattern predicted by the ‘Reward’ model. We concluded that the transformation of reward in a trial by trial m [file pone.0195399.s007.pdf]

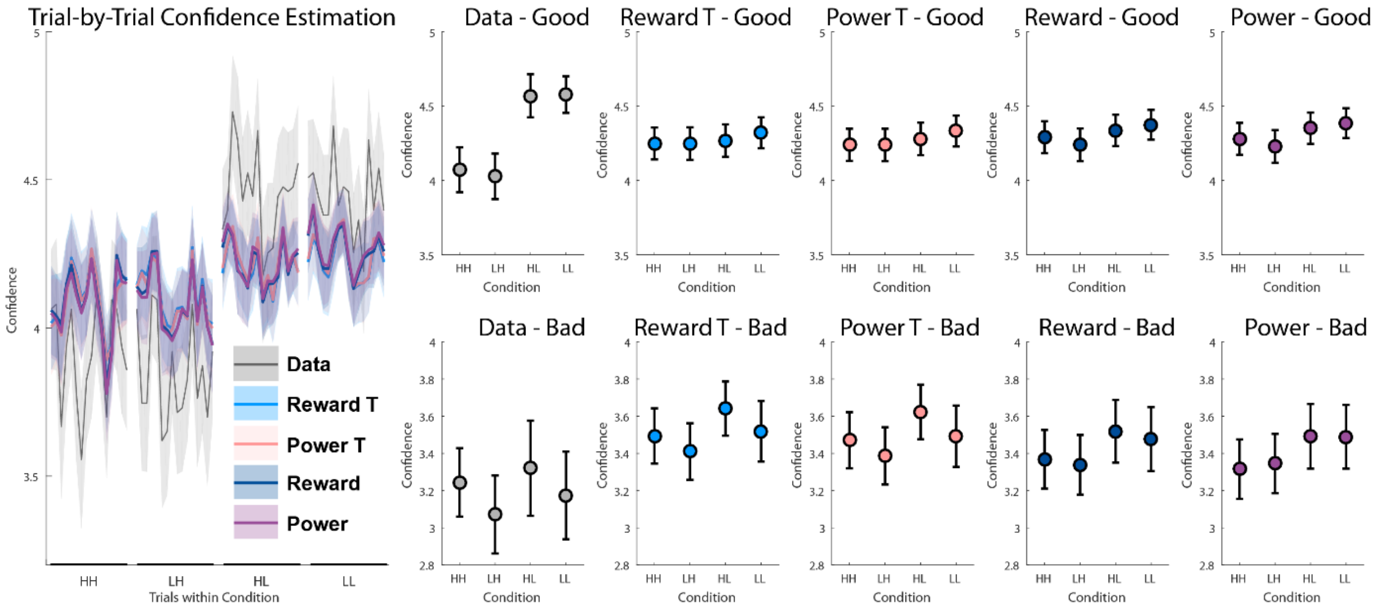

S7 Fig. Power Utility Model Performance in Experiment 1

To examine other utility functions we fitted a utility model which transforms the rewards in each trial according to the power utility function [1,2]:

$$\mathrm{U(R)} = \frac{R^{(1-\gamma)}}{1-\gamma} \quad 0 \le \gamma < 1$$

Where γ is the risk aversion factor – the closer it is to 1 the participant is more risk averse (and the closer the function is to log(r)). We used a model that learns from these transformed values, i.e. from utilities and not directly from the rewards, but was otherwise exactly the same as the 'Reward' model:

$$\begin{cases} Q_a(t+1) = Q_a(t) + \alpha\left(U\left(R(t)\right) - Q_a(t)\right) \\ \qquad\qquad Q_b(\mathrm{t}+1) = \mathrm{Q}_b(t) \end{cases}$$

When option a is chosen, its value is updated according to the difference between its current value and the utility of the option's current reward, with a learning rate α. Decision in each trial was then carried using a softmax rule.

We fitted this 'Power' model to the choice data, and an additional 'Power-T' model which added the drift to threshold of the unchosen option mechanism:

$$\begin{cases} Q_a(t+1) = Q_a(t) + \alpha\left(U\left(R(t)\right) - Q_a(t)\right) \\ \quad Q_b(\mathrm{t}+1) = \mathrm{Q}_b(t) + \alpha\left(T - Q_b(t)\right) \end{cases}$$

We examined the fit of these models to the data and how well they predicted the confidence reports.

We found that the ‘Power’ and ‘Power-T’ models performed very similarly to the ‘Reward’ and ‘Reward-T’ models respectively. Their WAIC values were: ‘Power’ 228.06 ± 67.46, ‘Power-T’: 214.51 ± 68.66, whereas the ‘Reward’ models WAIC values were: ‘Reward’ 226.55 ± 67.67, ‘Reward-T’ 214.57 ± 68.79. ‘Power-T’ was as good as our best model in explaining choice behaviour.

We than examined how well the ‘Power’ models explained confidence reports. Again, they fared similarly to the ‘Reward’ models with linear fit (R^2) of: Power’ 0.21 ± 0.22, ‘Power-T’: 0.21 ± 0.21, whereas the ‘Reward’ models WAIC values were: ‘Reward’ 0.21 ± 0.22, ‘Reward-T’ 0.21 ± 0.21.

Finally, we examined the predicted confidence reports in the four condition blocks, and found that the patterns predicted by the ‘Power-T’ model were identical to the pattern predicted by the ‘Reward’ model. We concluded that the transformation of reward in a trial by trial manner did not introduce any new mechanism to learning beyond the one already implemented by the ‘Reward’ model.
