# Supplementary material for: Stochastic satisficing account of confidence in uncertain value-based decisions
Source: PLoS One. 2018 Apr 5;13(4):e0195399. doi: 10.1371/journal.pone.0195399 (PMC5886535; doi:10.1371/journal.pone.0195399)
Supplement: S8 Fig — We followed the same optimality analysis as in S3 Fig with the reward distributions from experiment 2, and varied the acceptability threshold (blue to yellow lines). We found that the models varied dramatically in the relative values they assigned the options. Again, a greedy decision maker would therefore be able to accumulate similar amount of rewards using each model. However, a noisy decision maker (modeled using softmax) may be more likely to choose the low mean reward option in some conditions, according to the models’ predictions. (PDF) [file pone.0195399.s008.pdf]

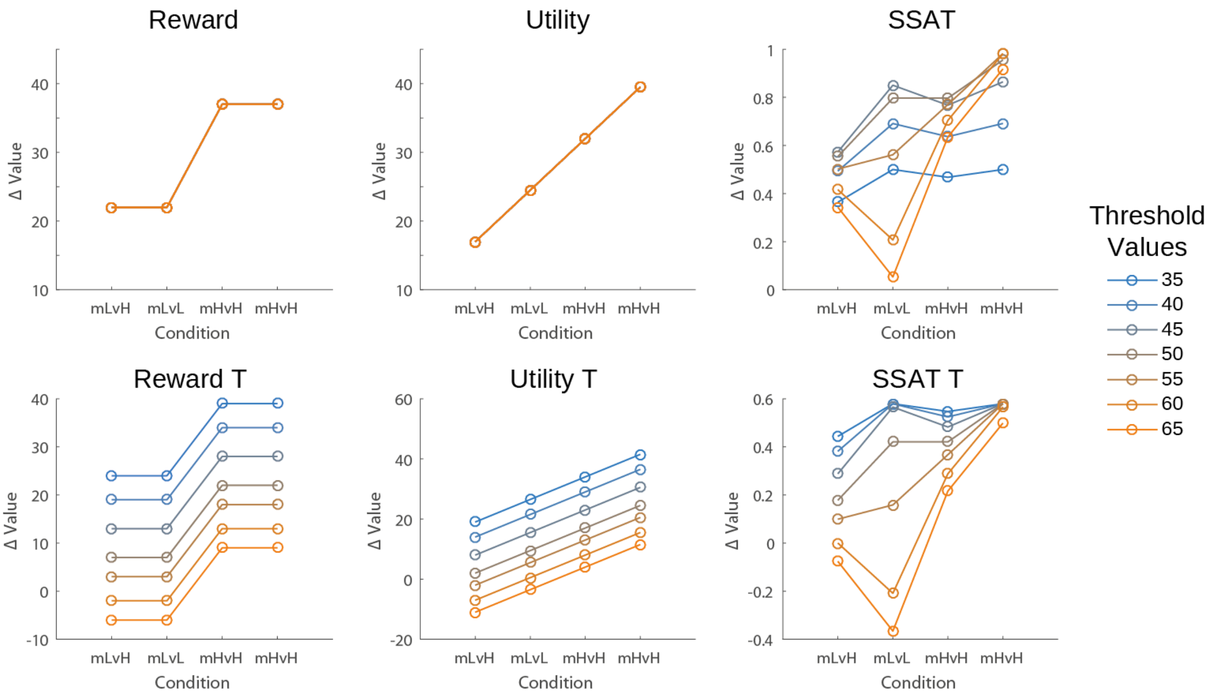


**S8 Fig. Value-Differences Optimality Analysis in Experiment 2**

We followed the same optimality analysis as in Figure S3 with the reward distributions from experiment 2, and varied the acceptability threshold (blue to yellow lines). We found that the models varied dramatically in the relative values they assigned the options. Again, a greedy decision maker would therefore be able to accumulate similar amount of rewards using each model. However, a noisy decision maker (modeled using softmax) may be more likely to choose the low mean reward option in some conditions, according to the models' predictions.
