# Supplementary material for: Stochastic satisficing account of confidence in uncertain value-based decisions
Source: PLoS One. 2018 Apr 5;13(4):e0195399. doi: 10.1371/journal.pone.0195399 (PMC5886535; doi:10.1371/journal.pone.0195399)
Supplement: S9 Fig — (A) Trial-by-Trial frequency of choosing the good option across participants (grey line) and models estimations of probability of choosing the good option (coloured lines), averaged across participants (shaded areas represent SEM). (B) Models’ estimations were averaged between trials 10–25 in each block. The average choices made by participants is displayed in grey. Most models were able to capture the pattern of the participants’ choice behaviour. overall best fitting model, across all trials, was the ‘Reward T’. (PDF) [file pone.0195399.s009.pdf]

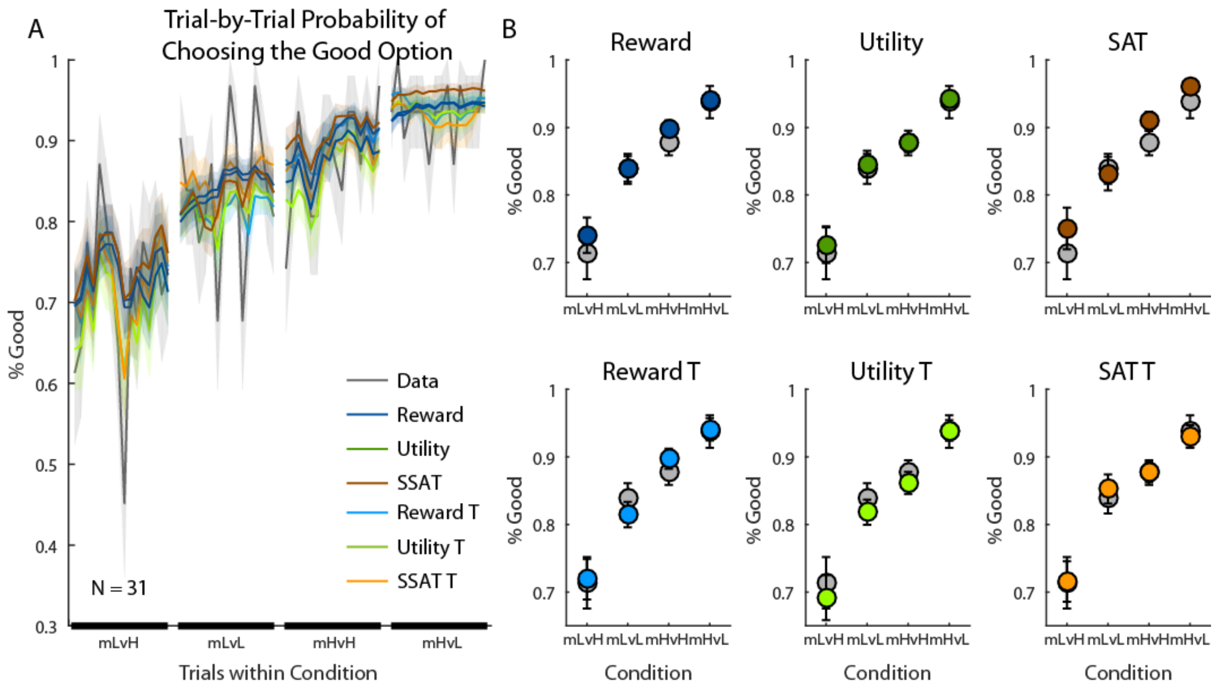

S9 Fig. Models fit to choices in experiment 2

(A) Trial-by-Trial frequency of choosing the good option across participants (grey line) and models estimations of probability of choosing the good option (coloured lines), averaged across participants (shaded areas represent SEM). (B) Models' estimations were averaged between trials 10-25 in each block. The average choices made by participants is displayed in grey. Most models were able to capture the pattern of the participants' choice behaviour. overall best fitting model, across all trials, was the 'Reward T'.
