# Supplementary material for: Stochastic satisficing account of confidence in uncertain value-based decisions
Source: PLoS One. 2018 Apr 5;13(4):e0195399. doi: 10.1371/journal.pone.0195399 (PMC5886535; doi:10.1371/journal.pone.0195399)
Supplement: S10 Fig — We compared the individual parameters estimated for each of our drift (and best performing) models. The Reward-T and SSAT-T models’ parameters for learning rates and threshold were almost identical for all our participants. Reward model gave the best fit to decisions, while the SSAT-T model gave the best fit to confidence reports. This indicates that these models may use a shared mechanism for decisions, but the SSAT-T model uses the reward variance information to generate confidence reports. Parameters estimations were not as similar for the SSAT-T and the Utility-T. (PDF) [file pone.0195399.s010.pdf]

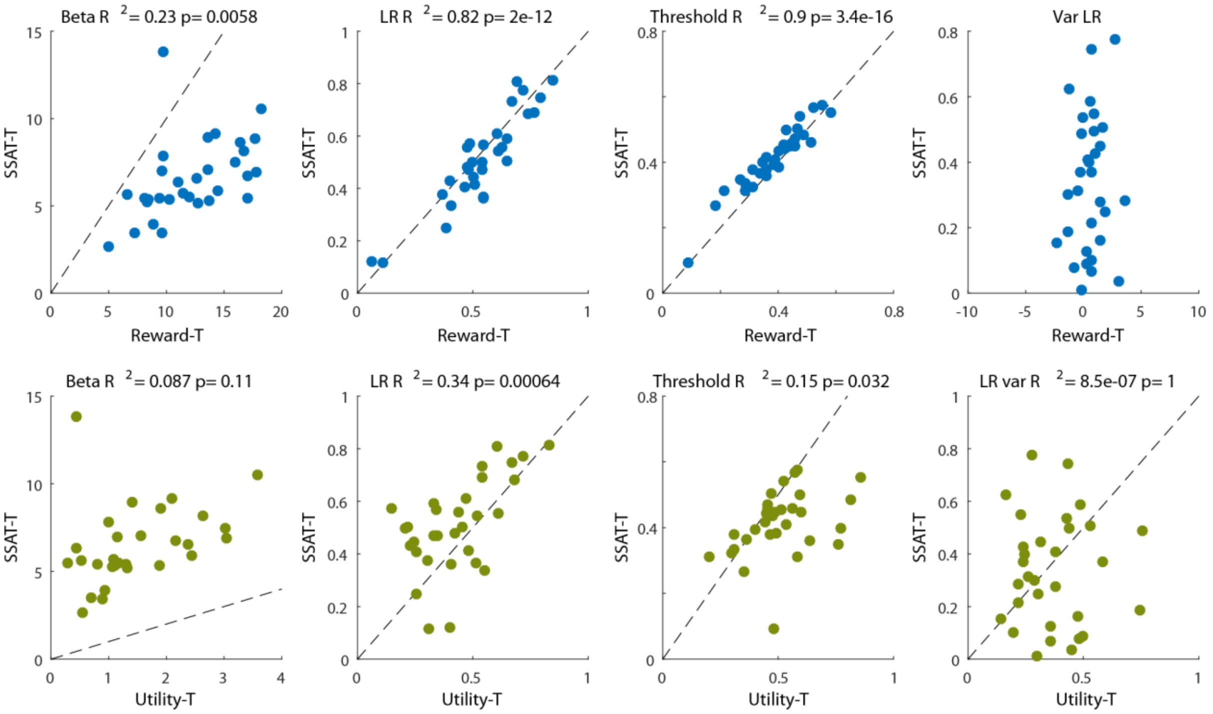

S10 Fig. Relations between estimated parameters in different models in experiment 2.

We compared the individual parameters estimated for each of our drift (and best performing) models. The Reward-T and SSAT-T models' parameters for learning rates and threshold were almost identical for all our participants. Reward model gave the best fit to decisions, while the SSAT-T model gave the best fit to confidence reports. This indicates that these models may use a shared mechanism for decisions, but the SSAT-T model uses the reward variance information to generate confidence reports. Parameters estimations were not as similar for the SSAT-T and the Utility-T.
